# Exogenous Aβ seeds induce Aβ depositions in the blood vessels rather than the brain parenchyma, independently of Aβ strain-specific information

**DOI:** 10.1186/s40478-021-01252-0

**Published:** 2021-09-10

**Authors:** Tsuyoshi Hamaguchi, Jee Hee Kim, Akane Hasegawa, Ritsuko Goto, Kenji Sakai, Kenjiro Ono, Yoshinori Itoh, Masahito Yamada

**Affiliations:** 1 Department of Neurology and Neurobiology of Aging, Kanazawa University Graduate School of Medical Sciences, 13-1 Takara-machi, Kanazawa, 920-8640 Japan; 2 Department of Internal Medicine, Division of Neurology, Showa University School of Medicine, Tokyo, Japan; 3 Yokufukai Geriatric Hospital, Tokyo, Japan; 4 Department of Internal Medicine, Division of Neurology, Kudanzaka Hospital, Tokyo, Japan

**Keywords:** Amyloid β peptide, Alzheimer’s disease, Cerebral amyloid angiopathy, Transmission, Strain, Iatrogenic

## Abstract

Little is known about the effects of parenchymal or vascular amyloid β peptide (Aβ) deposition in the brain. We hypothesized that Aβ strain-specific information defines whether Aβ deposits on the brain parenchyma or blood vessels. We investigated 12 autopsied patients with different severities of Aβ plaques and cerebral amyloid angiopathy (CAA), and performed a seeding study using an Alzheimer’s disease (AD) mouse model in which brain homogenates derived from the autopsied patients were injected intracerebrally. Based on the predominant pathological features, we classified the autopsied patients into four groups: AD, CAA, AD + CAA, and less Aβ. One year after the injection, the pathological and biochemical features of Aβ in the autopsied human brains were not preserved in the human brain extract-injected mice. The CAA counts in the mice injected with all four types of human brain extracts were significantly higher than those in mice injected with PBS. Interestingly, parenchymal and vascular Aβ depositions were observed in the mice that were injected with the human brain homogenate from the less Aβ group. The Aβ and CAA seeding activities, which had significant positive correlations with the Aβ oligomer ratio in the human brain extracts, were significantly higher in the human brain homogenate from the less Aβ group than in the other three groups. These results indicate that exogenous Aβ seeds from different Aβ pathologies induced Aβ deposition in the blood vessels rather than the brain parenchyma without being influenced by Aβ strain-specific information, which might be why CAA is a predominant feature of Aβ pathology in iatrogenic transmission cases. Furthermore, our results suggest that iatrogenic transmission of Aβ pathology might occur due to contamination of brain tissues from patients with little Aβ pathology, and the development of inactivation methods for Aβ seeding activity to prevent iatrogenic transmission is urgently required.

## Introduction

Alzheimer’s disease (AD) is the most frequent cause of dementia, and senile plaques with extracellular deposition of amyloid β peptides (Aβ) and intracellular neurofibrillary tangles (NFTs) with abnormally phosphorylated tau protein are pathological hallmarks of AD [28]. Extracellular Aβ deposition is considered the primary pathological event in AD, because all genetic mutations in patients with inherited AD have been found in genes related to Aβ [55]. However, NFTs, and not Aβ deposition, are significantly correlated with the progression of cognitive impairment [2].

Cerebral amyloid angiopathy (CAA) is a cerebrovascular amyloid deposition that causes normotensive cerebral haemorrhage in older individuals [63, 65]. Seven amyloid proteins that involve the vessels of patients with CAA have been reported, with Aβ being the most common amyloid protein in patients with CAA [63, 65]. According to previous pathological studies, CAA is observed in approximately half of elderly individuals, and the incidence of CAA increases with age [54, 63, 65, 66]. CAA is also common in AD patients, and the prevalence of CAA is 80%–90% in patients with AD [63, 65, 66]. Aβ is a 36–43 amino acid peptide cleaved from the amyloid precursor protein [8] by β-secretase and γ-secretase, and senile plaques mainly consist of 42-residue peptides (Aβ42), whereas Aβ in CAA is mainly a 40-residue variant (Aβ40) [63, 65]. Aβ40 does not aggregate as easily as Aβ42, whereas Aβ42 promptly deposits in the brain parenchyma as senile plaques. Aβ40 is considered to be cleared from the cerebral cortex through intramural periarterial drainage (IPAD) and glymphatic drainage pathways, in which Aβ40 aggregates on vascular basement membranes [1, 3, 52, 61]. Although most patients with CAA have accompanying parenchymal Aβ deposition, there have been reports of pure CAA patients with fewer of Aβ depositions or NFTs in the brain parenchyma, as well as AD patients presenting without CAA [33, 57, 59]. The precise reason why Aβ deposits in the brain parenchyma or blood vessels has not been elucidated.

Recently, under experimental settings, it has been established that neurodegenerative disorders characterized by the deposition of aberrant proteins, such as Aβ and tau, in the brain can propagate to different cells via a prion-like mechanism, as well as to different regions within an individual and to different individuals [32]. In prion diseases, the pathological and physicochemical features of prion protein (PrP) are maintained among individuals by strains of abnormal PrP (PrP^Sc^) [10, 11]. Several studies have shown that differences in the molecular conformation of Aβ correlate with pathological phenotypes of Aβ pathology in the brains of AD mouse models [24, 51, 56, 60], which suggests that Aβ has several strains, similar to PrP^Sc^.

Mutations in the amyloid precursor protein (APP) gene (*APP*) located around β- and γ-secretase cleavage sites cause familial AD [7]. On the other hand, some mutations in the middle of *APP*, such as E693Q for hereditary cerebral haemorrhage with amyloidosis (HCHWA)-Dutch type [42, 44] and E693K for HCHWA-Italian type [6], cause severe CAA with few parenchymal Aβ depositions and NFTs. In HCHWA-Dutch patietns, the mutation of E693Q mutation leads to overproduction of Aβ40, and an experimental study using a HCHWA-Dutch mouse model showed that an increase in the ratio of Aβ40/Aβ42 in the brain homogenate plays an important role in the deposition of Aβ on brain blood vessels [26, 27]. Different distributions of Aβ pathology among different point mutations of *APP* would show that structural differences of Aβ, in addition to the ratio of Aβ40/Aβ42, might contribute to whether Aβ deposits in brain parenchyma or vessels, which suggests that Aβ strain-specific information would determine the distribution of Aβ pathology.

We hypothesized that Aβ strain-specific information defines whether Aβ deposits in brain parenchyma or blood vessels in sporadic cases. To this end, in the present study, we performed a seeding study using an AD mouse model in which brain homogenates derived from patients with different severities of Aβ plaques and CAA were injected intracerebrally. We then evaluated the pathological and biochemical features of the AD mouse model one year after injection.

## Materials and methods

### Autopsied patients

We included 12 autopsied patients who died at Yokufukai Geriatric Hospital. All brain samples were collected from donors for whom written informed consent for the autopsy, as well as use of samples and clinical information for research purposes, were obtained. This study was approved by the institutional ethics committee of Kanazawa University (1276).

### Genetic analysis

Genomic DNA extracted from patients’ blood or frozen brains was used to analyze the polymorphism of the apolipoprotein E gene (*APOE*) [29].

### Neuropathology of the autopsied human brains

Five-µm-thick, formalin-fixed, paraffin-embedded tissue blocks of all patients were sectioned. Postmortem delay was generally less than 24 h. Brain sections were stained with hematoxylin–eosin, Klüver-Barrera, Congo red, and Gallyas-Braak. Single-labelling immunohistochemical studies were performed on sections of the left frontal, parietal, temporal, and occipital lobes using antibodies against Aβ_17–24_ (4G8, 1:5,000; Covance), Aβ_35–40_ (1A10, 1:1,000; Immuno-Biological Laboratory), Aβ_1–42_ (1:100; Immuno-Biological Laboratory), and phosphorylated tau (AT8, 1:1,000; Innogenetics) using the avidin–biotin-peroxidase complex (ABC) method (Vector) with diaminobenzidine as the chromogen. According to the manufacturer’s datasheets, antibodies against Aβ_35–40_ and Aβ_1–42_ are human Aβ40 specific (https://www.ibl-japan.co.jp/en/search/product/detail/id=3520) and human Aβ42 specific (https://www.ibl-japan.co.jp/en/search/product/detail/id=3720), respectively. Neuropathological analysis was performed by a scientist (KS) blinded to the clinical information of the patients. Neuropathological diagnosis was made using the Consortium to Establish a Registry for AD (CERAD) score [46], Thal phase [58], Braak AT8 stage [5], and CAA score [43]. Based on the pathological features, we classified the autopsied patients into four groups: AD, CAA, AD + CAA, and less Aβ.

### Quantification of Aβ pathology of the autopsied human brains

All quantification was performed by a scientist (AH) blinded to the clinical information of the patients. The Aβ loads (% positive area stained with 4G8), Aβ40 loads (% positive area stained with 1A10), and Aβ42 loads (% positive area stained with anti-Aβ_1–42_ antibody) in the cortices of the frontal, parietal, temporal, and occipital lobes were quantified in 30 images per lobe (120 images per one patient) that were captured with a 20 × objective under a bright field using an Olympus BX-51 microscope, Olympus DP71 digital camera, and WinROOF® custom-designed software (Mitani Corporation. Fukui, Japan).

### Preparation of the human brain tissue extracts

The right occipital lobe from all autopsied patients was frozen on dry ice and stored at − 80 °C until the experiment. The brain samples were homogenized at 10% (w/v) in PBS, vortexed, sonicated 3 × 5 s and centrifuged at 3000 × g for 5 min. The supernatant was divided into aliquots and frozen (human brain extracts). The PBS-insoluble pellet was sonicated in 5 M guanidine and 50 mM Tris–HCl of the same volume as PBS, solubilized by agitation at room temperature (around 25 °C) for 30 min, and frozen (human brain pellets).

### Seeding study using transgenic mice

Homozygous R1.40 APP-transgenic mice (R1.40 mice), which carry a yeast artificial chromosome containing the entire genomic copy of human APP harbouring the “Swedish” familial AD double mutation (APPK670N-M671L) [40], were transferred from Tübingen University to Kanazawa University, and were generated by breeding the homozygous offspring. All animal studies were approved by the Institutional Animal Experiment Committee of Kanazawa University (AP-132607), and performed in accordance with the Guidelines for the Care and Use of Laboratory Animals at Kanazawa University (Kanazawa, Japan). A neuropathological study showed that both diffuse and cored plaques, as well as CAA, were observed in the brains of aged R1.40 mice [39].

Human brain extracts were injected into the brains of the R1.40 mice, as previously reported [19]. Three-month-old homozygous R1.40 mice were anaesthetized with a mixture of ketamine/xylazine anaesthesia (ketamine 100 mg/kg and xylazine 10 mg/kg), and bilateral stereotaxic injections of human brain extract were infused into the hippocampus (2.5 μl) and overlying neocortex (1.0 μl) (AP − 2.5 mm, L ± 2.0 mm, DV − 1.0/− 1.8 mm). The surgical area was cleaned with sterile saline, the incision was sutured, and the mice were monitored until recovery from anaesthesia.

### Quantification of the Aβ pathology of the R1.40 mice brains

The 15-month-old R1.40 mice were perfused transcardially with PBS under deep ketamine/xylazine anaesthesia (ketamine 400 mg/kg and xylazine 40 mg/kg) one year after the injection of the human brain extract. The brains were harvested, hemidissected, and the left hemispheres were fixed in 4% paraformaldehyde for 24 h for histological studies, while the right hemispheres were frozen rapidly in liquid nitrogen and stored at − 80 °C for biochemical studies.

For assessing of Aβ deposition in the brains of the R1.40 mice, 4% paraformaldehyde-fixed, paraffin-embedded left hemispheres were sectioned in the coronal plane using a microtome at a thickness of 5 µm. Sections were routinely deparaffinized and hydrated in a graded series of ethanol, pre-treated with 99% formic acid for 5 min, and immersed in 0.3% hydrogen peroxide and methanol for 30 min to block endogenous peroxidase before preblocking at ambient temperature with serum-free protein block (Dako, Glostrup, Denmark). Aβ immunohistochemical staining was performed using antibodies against Aβ_17–24_ (4G8, 1:5,000), Aβ_35–40_ (1A10, 1:1,000), and Aβ_1–42_ (1:100) in conjunction with the Liquid DAB Substrate Chromogen System (Dako. Glostrup, Denmark). Aβ loads (% positive area stained with 4G8), Aβ40 loads (% positive area stained with 1A10), and Aβ42 loads (% positive area stained with anti-Aβ_1–42_ antibody) in the brain were quantified under a bright field using a Keyence BZ-700 microscope (Keyence Corporation, Osaka, Japan) and analyzed using a BZ-X analyzer (Keyence). The number of 4G8-positive blood vessels per 1 mm^2^ was also investigated (CAA counts). In total, five coronal sections were assessed by a scientist (JHK) who was blinded to the profile of each section of the mouse brain.

### Preparation of the R1.40 mice brain tissue homogenates

The frozen R1.40 mouse brain samples were homogenized at 10% (w/v) in PBS, vortexed, sonicated for 3 × 5 s, and centrifuged at 3000 × g for 5 min. The supernatant was divided into aliquots and then frozen (mouse brain extracts). The PBS-insoluble pellet was sonicated in 5 M guanidine and 50 mM Tris–HCl of the same volume as PBS, solubilized by agitation at room temperature (around 25 °C) for 30 min, and frozen (mouse brain pellets).

### Quantitative assessment of Aβ in the brain

To quantitatively assess Aβ in the brain, a sandwich enzyme-linked immunosorbent assay (ELISA) was used to detect Aβ_1–40_ (Human βAmyloid (1–40) ELISA Kit *Wako* II; FUJIFILM Wako Pure Chemical Corporation, Osaka, Japan) and Aβ_1–42_ (Human βAmyloid (1–42) ELISA Kit *Wako*, High-Sensitive; FUJIFILM Wako Pure Chemical Corporation, Osaka, Japan) in the human brain extracts, human brain pellets, mouse brain extracts, and mouse brain pellets according to the manufacturer’s instructions.

### Analysis of A11-positive oligomers in the human brains

To investigate A11-positive oligomers in the human brain extracts, dot blot assays were performed as described previously [21]. Human brain extracts were applied directly to a nitrocellulose membrane and air-dried, and the membrane was probed with an A11 antibody (1/1000, StressMarq Biosciences Inc. Victoria, Canada), which recognizes oligomers but not monomers or fibrils of several proteins that form amyloid, including Aβ [18, 37]. Immunoreactivity was quantified densitometrically using a LAS-4000 mini and Multi Gaurge Ver.3.X (Fujifilm, Tokyo, Japan). A11-positive Aβ oligomers ranged in size from approximately tetramers to 20-mers [36]. Each sample was analyzed three times, and the values of A11-positive oligomers were indicated by each optical density divided by the average of all optical densities (value of A11-positive oligomers). The ratio of A11-positive oligomers in the human brain extract was calculated, and the value of A11-positive oligomers was divided by the concentration of Aβ40 + Aβ42 (A11-positive oligomer ratio).

### Analysis of high molecular weight Aβ oligomers in the human brains

We investigated high molecular weight (HMW) Aβ oligomers in the human brain extracts using an ELISA kit (High Molecular Amyloid β Oligomer ELISA Kit *Wako* Ver. 2; FUJIFILM Wako Pure Chemical Corporation, Osaka, Japan) according to the manufacturer’s instructions. This ELISA kit can detect mainly 10–20-mers of Aβ oligomers, and the measured value is the value calculated based on a 16-mer multiple-antigenic peptides [17, 34]. The ratio of HMW Aβ oligomers in the Aβ40 and Aβ42 monomers was calculated by dividing the concentration of HMW Aβ oligomers by the consentrations of Aβ40 + Aβ42 (HMW Aβ oligomer ratio).

### Calculation of Aβ and CAA seeding activity in human autopsied patients

The induction of Aβ pathology in the brain extracts is dependent on the amount of Aβ when the incubation period is the same [45]. We calculated the Aβ and CAA seeding activities of the human brain extracts from autopsied patients as follows: the Aβ load and CAA counts in the human brain extract-injected R1.40 mice were divided by the concentrations of Aβ40 + Aβ42 in the human brain extracts, as in a previous study [67].

### Proteinase K treatment of the human and mouse brain extracts

The human and mouse brain extracts were treated with increasing concentrations (0, 25, 50, and 100 µg/mL) of proteinase K (PK) (Nacalai Tesque, Inc., Kyoto, Japan) for 1 h at 37 °C. PK treatment was blocked by adding NuPage LDS Sample Buffer (Thermo Fisher Scientific, Inc., Massachusetts, USA) and NuPage Sample Reducing Agent (Thermo Fisher Scientific, Inc.), and then incubated for 10 min at 70 °C. The samples were analyzed by Western blotting using NuPage 4–12% Bis–Tris Gel using NuPage MES running buffer (Thermo Fisher Scientific, Inc.) and antibodies against Aβ_1–16_ (6E10, 1:2,000; BioLegend) as the primary antibodies.

## Statistical analysis

All values are expressed as means ± standard deviation (SD). Differences in Aβ loads, Aβ40 loads, Aβ42 loads, and CAA counts, concentrations of Aβ40, Aβ42, and Aβ40 + Aβ42, and Aβ40/Aβ42 ratios in mouse brain extracts and mouse brain pellets were compared among the five groups: the R1.40 mice injected with human brain extracts from the patients in the AD group (R1.40 mice-AD), R1.40 mice injected with human brain extracts from the patients in the CAA group (R.140 mice-CAA), R1.40 mice injected with human brain extracts from the patients in the AD + CAA group (R.140 mice-AD + CAA), R1.40 mice injected with human brain extracts from the patients in the less Aβ group (R1.40 mice-less Aβ), and R1.40 mice injected with PBS (R1.40 mice-PBS). The differences were analyzed using one-way ANOVA followed by a Bonferroni post hoc analysis. Differences among the groups of autopsied patients in terms of Aβ and CAA seeding activity were analyzed using a one-way ANOVA followed by a Bonferroni post hoc analysis. Correlations between Aβ seeding activity and A11-positive oligomer ratios, Aβ seeding activity and HMW Aβ oligomer ratios, CAA seeding activity and A11-positive oligomer ratios, and CAA seeding activity, and HMW Aβ oligomer ratios were analyzed using Pearson’s correlation tests. Statistical significance was defined as *P* < 0.05. Statistical analyses were performed using IBM SPSS Statistics version 25 (SPSS Japan Inc. Tokyo, Japan).

## Results

### Neuropathological findings of autopsied patients

A summary of the 12 autopsied patients in the present study is shown in Table 1. Based on the pathological features, all patients were classified into four groups as follows: AD group (Patients 1–3), CAA group (Patients 4–6), AD + CAA group (Patients 7–10), and less Aβ group (Patients 11 and 12). The ages at death were 79–92 years old, and 10 patients were analyzed for the *ApoE* genotype; seven patients were *ApoE* 3/3, two patients were *ApoE* 3/4, and one patient was *ApoE* 2/3 (Table 1).

**Table 1:** Summary of autopsied patients

Pt	Age at death (yrs)	Sex	*ApoE* genotype	CERAD score	Thal phase	Braak AT8 stage	CAA score	Type
1	79	male	3/4	C	5	5	2	AD
2	87	female	ND	C	5	5	1	AD
3	92	female	ND	B	5	5	0	AD
4	85	male	2/3	0	2	2	4	CAA
5	87	female	3/3	0	5	2	8	CAA
6	91	male	3/4	A	5	3	8	CAA
7	81	female	3/3	C	5	4	8	AD + CAA
8	89	male	3/3	B	5	5	8	AD + CAA
9	80	female	3/3	B	5	5	8	AD + CAA
10	89	female	3/3	C	5	5	8	AD + CAA
11	86	female	3/3	0	3	2	1	less Aβ
12	82	male	3/3	0	1	2	2	less Aβ

Pt., patients; yrs, year; *ApoE*, apolipoprotein E; ND, not done; AD, Alzheimer’s disease; CAA, cerebral amyloid angiopathy; Aβ, amyloid β protein

**Fig. 1:**
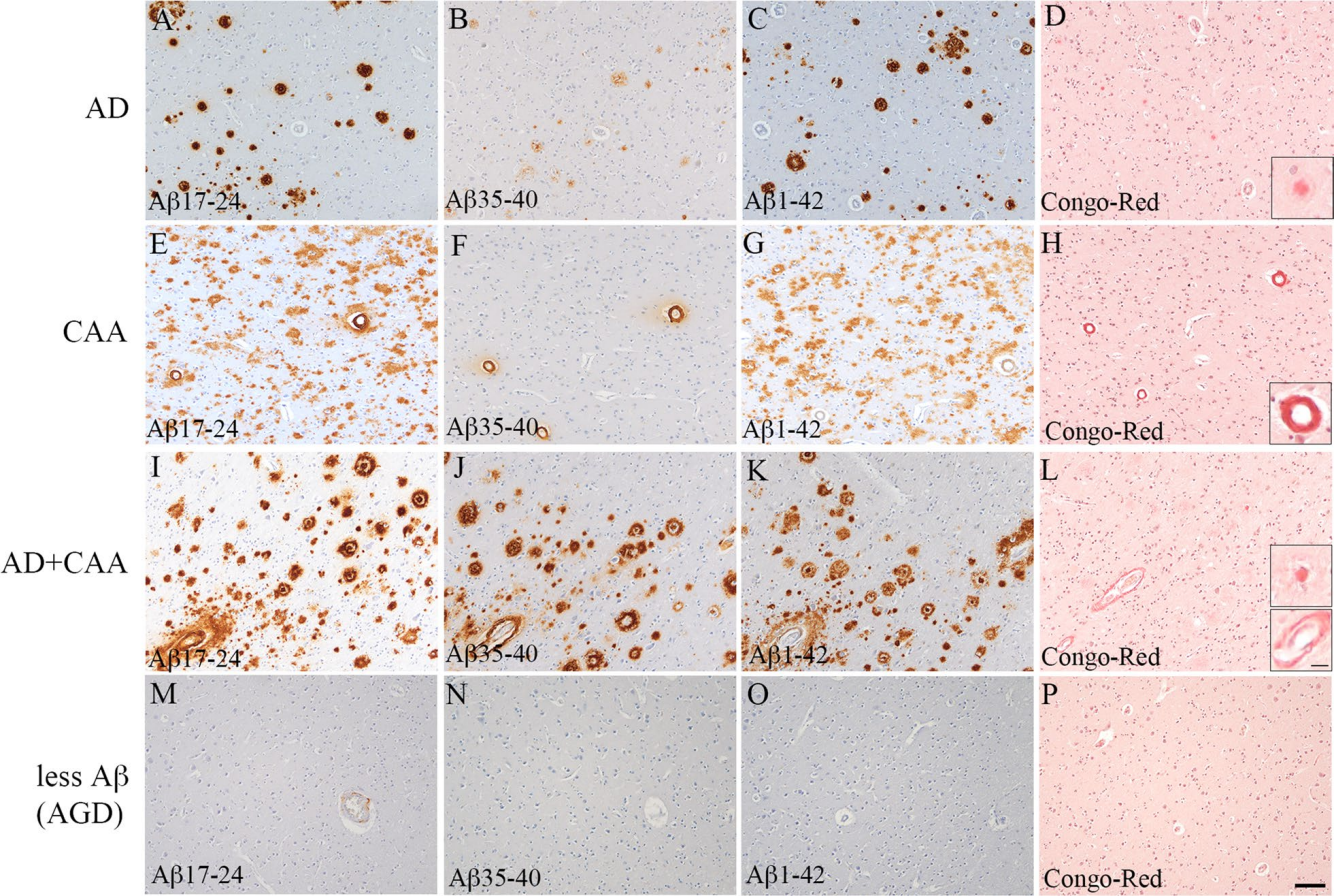
Representative images of the amyloid pathology of the autopsied human brains. The images of the left occipital lobe belong to groups with Alzheimer’s disease (AD) (**A**–**D**), cerebral amyloid angiopathy (CAA) (**E**–**H**), AD + CAA (**I**–**L**), and less amyloid β peptide (Aβ) pathology (**M**–**P**). Antibodies against Aβ_17–24_ (4G8, 1:5,000) (**A**,** E**,** I**, and** M**), Aβ_35–40_ (1A10, 1:1,000) (**B**,** F**,** J**, and** N**), and Aβ_1–42_ (1:100) (**C**, **G**,** K**, and** O**) were used as primary antibodies for immunohistochemistry, and Congo red staining was also performed (**D**,** H**, **L**, and** P**). In the AD group, many cored and diffuse plaques with little CAA were observed, and the majority of the Aβ pathologies consisted of Aβ42 (**A**–**C**). The cored plaques were stained with Congo red and displayed apple-green birefringence in polarized light, but diffuse plaques were not stained with Congo red (**D**). The small insert shows a Congo red-positive plaque with high magnification (**D**). In the CAA group, many brain vessels with CAA anad many diffuse plaques were observed despite the absence of cored plaques (**E**–**G**). CAA consisted of both Aβ40 and Aβ42, and diffuse plaques were stained mainly with Aβ42 (**F** and **G**). The vessels with CAA were positive for Congo red, but there were almost no lesions in the brain parenchyma (**H**). The small insert shows a Congo red-positive vessel at high magnification (**H**). In the AD + CAA group, both cored and diffuse plaques in the grey matter and many vessels with CAA were observed, and the majority of cored and diffuse plaques were stained with Aβ42 rather than Aβ40 (**I**-**K**). CAA consisted of both Aβ40 and Aβ42 (**J** and** K**). The cored plaques and vessels with CAA were stained with Congo red (**L**). The small insert shows Congo red-positive plaque and vessel at high magnification (**L**). In the less Aβ group, few diffuse plaques and vessels with CAA were observed (M–P). The scale bar represents 100 µm, and 25 µm for small inserts. AGD: argyrophilic grain disease

Neuropathological studies of Aβ pathology in representative patients are shown in Fig. 1. In the AD group, many cored and diffuse plaques were observed in the cerebral cortices, whereas CAA was hardly detected (Fig. 1a–c). Most Aβ pathologies consisted of Aβ42 (Fig. 1c), but little Aβ40 pathology was observed (Fig. 1b). The cored plaques were stained with Congo red and displayed apple-green birefringence in polarized light, but diffuse plaques were not stained with Congo red (Fig. 1d). The CERAD score was C in two patients and B in another patient, the Thal phase was five in all three patients, and the CAA scores ranged from 0 to 2 (Table 1). In the CAA group, we found many brain vessels with CAA as well as many diffuse plaques, although few cored plaques were observed (Fig. 1e–g). CAA consisted of both Aβ40 and Aβ42 (Fig. 1f and g), and diffuse plaques were stained mainly with Aβ42 (Fig. 1g). The vessels with CAA were stained with Congo red, but almost no stained lesions were observed in the brain parenchyma (Fig. 1h). The CERAD score was 0 in two patients and A in another patient, the Thal phase was 5 in two patients and 2 in another patient, and the CAA scores ranged from 4 to 8 (Table 1). In the AD + CAA group, we observed both cored and diffuse plaques in the grey matter as well as many vessels with CAA (Fig. 1i–k). Most of the cored and diffuse plaques were stained with Aβ42, not Aβ40 (Fig. 1j and k), and the CAA consisted of both Aβ40 and Aβ42 (Fig. 1j and k). The cored plaques and vessels with CAA were stained with Congo red (Fig. 1l). The CERAD score was B in two patients and C in the other two patients, the Thal phase was 5 in all four patients, and the CAA score was 8 in all four patients (Table 1). In the less Aβ group, small amounts of diffuse and cored plaques with slight CAA accumulation in the frontal and occipital lobes were observed in Patient 11. In Patient 12, there were few diffuse plaques and vessels with CAA (Fig. 1m–p). The CERAD score was 0 in all two patients, the Thal phase was 1 and 3, and the CAA score ranged from 1 to 2 (Table 1). We did not observe the pathological findings of the right occipital lobe that was used for the human brain extracts that were injected into the R1.40 mice brains.

**Fig. 2:**
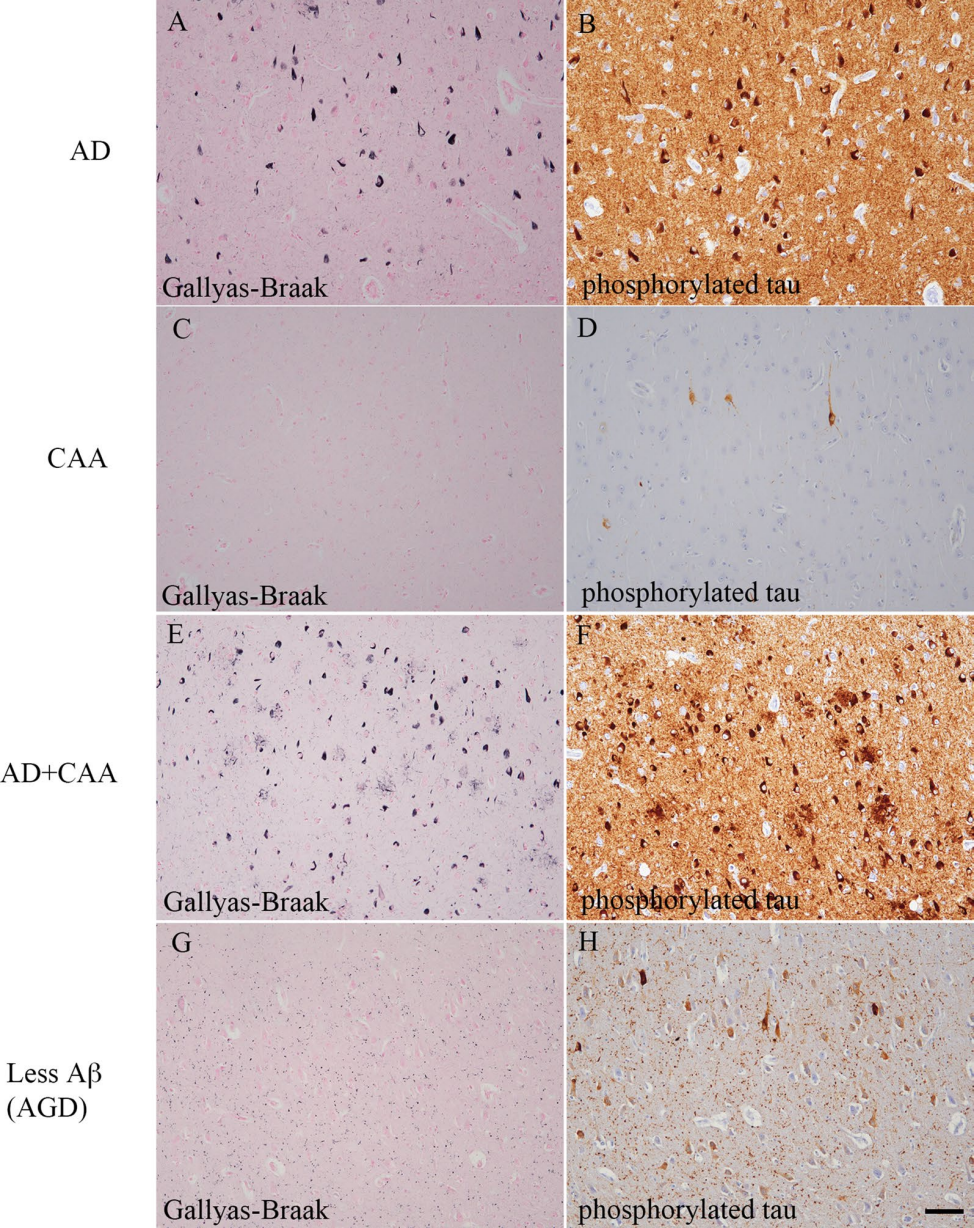
Representative images of the tau pathology of the autopsied human brains. The images of the left medial temporal lobe belong to groups with Alzheimer’s disease (AD) (**A** and B), cerebral amyloid angiopathy (CAA) (**C** and** D**), AD + CAA (**E** and **F**), and less amyloid β peptide (Aβ) pathology (G and H). Gallyas-Braak staining (**A**, **C**, **E**, and **G**) and immunohistochemistry using antibodies against phosphorylated tau (AT8, 1:1,000) as a primary antibody (**B**,** D**,** F**, and** H**) were performed. In the AD group, many neurofibrillary tangles (NFTs) and neuropil threads with dystrophic neurites were observed (**A** and** B**). In the CAA group, only a few pretangles were observed (**C** and** D**). In the AD + CAA group, many NFTs, neuropil threads and dystrophic neurites were observed (**E** and** F**). In the less Aβ group, many argyrophilic grains with some pretangles were observed (**G** and** H**). Scale bar represents 100 µm. AGD: argyrophilic grain disease

Neuropathological studies of tau pathology in representative patients are shown in Fig. 2. In the AD group, many neurofibrillary tangles (NFTs) and neuropil threads with dystrophic neurites were observed (Fig. 2a and b). In the CAA group, only a few pretangles were observed (Fig. 2c and d). In the AD + CAA group, many NFTs, neuropil threads and dystrophic neurites were observed (Fig. 2e and f). In the less Aβ group, many grains with some pretangles were observed (Fig. 2g and h). According to neuropathological studies, both of the 2 patients in the less Aβ group had argyrophilic grain disease (AGD).

**Fig. 3:**
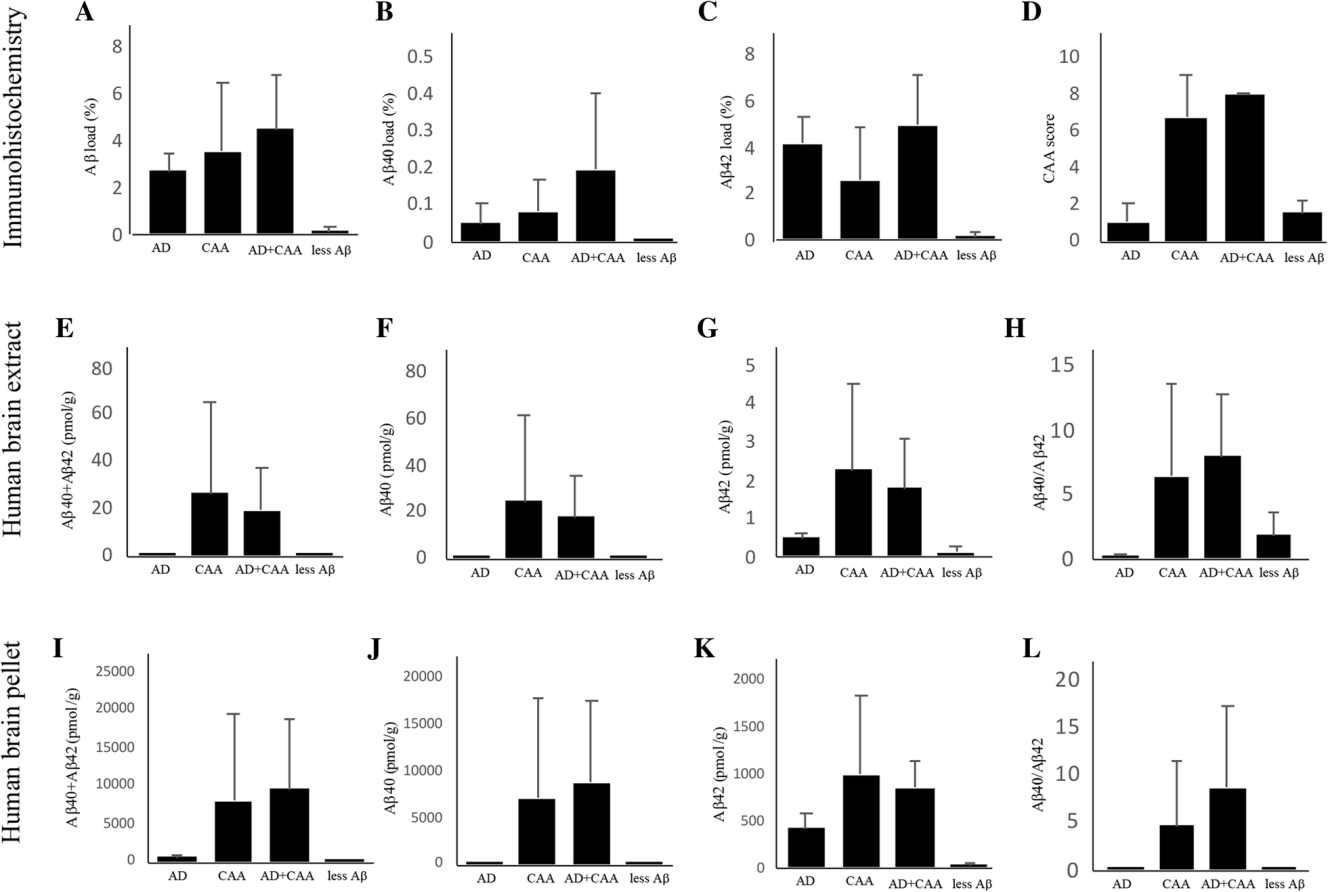
Quantitative analysis of amyloid β peptide (Aβ) pathology and biochemical studies of the autopsied patients. The images represent the quantitative analysis of Aβ pathology in the autopsied patients (**A**–**D**), and concentrations of Aβ40, Aβ42, and Aβ40 + Aβ42, as well as the ratio of Aβ40/Aβ42 in the human brain extracts (**E**–**H**) and human brain pellets (**I**–**L**). Compared to Aβ, the Aβ40 and Aβ42 loads in the Alzheimer’s disease (AD), cerebral amyloid angiopathy (CAA) and AD + CAA groups, those with less Aβ pathology were much lower (**A**–**C**). The CAA scores of the CAA and the AD + CAA groups were higher than those of the AD and the less Aβ groups (**D**). In the human brain extracts, concentrations of Aβ40 in the CAA and AD + CAA groups were much higher than those in the AD and less Aβ groups, and consequently the concentrations of Aβ40 + Aβ42 in the CAA and AD + CAA groups were also much higher (**E** and **F**). The concentrations of Aβ42 in the CAA and AD + CAA groups were also higher than those in the AD and less Aβ groups, but the differences were smaller than for the Aβ40 concentrations (**G**). The ratio of Aβ40/Aβ42 was Aβ42-dominant in the patients with AD group, while that of the other three groups was Aβ40 dominant (**H**). In the human brain pellets, the proportion of the concentrations of Aβ40 + Aβ42, Aβ40, and Aβ42, as well as the ratio of Aβ40/Aβ42 among the AD, CAA, AD + CAA, and less Aβ groups were similar to those in the human brain extracts (I–L)

A quantitative analysis of Aβ pathology in autopsied patients is shown in Fig. 3. In the less Aβ group, Aβ, Aβ40, and Aβ42 loads were much lower than those in the other three groups, but statistical analyses were not performed because of the small number of patients in each group (AD group, 3; CAA group, 3; AD + CAA group, 4; less Aβ group, 2). The CAA scores of the CAA and AD + CAA groups were higher than those of the AD and less Aβ groups (Fig. 3D).

### Concentration of Aβ in the human brain extracts and human brain pellets

In the human brain extracts, concentrations of Aβ40 in the CAA and AD + CAA groups were much higher than those in the AD and less Aβ groups, and consequently the concentrations of Aβ40 + Aβ42 in the CAA and AD + CAA groups were also much higher (Fig. 3e and f). The concentrations of Aβ42 in the CAA and AD + CAA groups were also higher than those in the AD and less Aβ groups, but the differences were smaller than for the concentrations of Aβ40 (Fig. 3g). The ratio of Aβ40/Aβ42 was Aβ42-dominant in the patients with AD group, while it was Aβ40 dominant in the other three groups (Fig. 3h).

In the human brain pellets, the proportion of the concentrations of Aβ40 + Aβ42, Aβ40, and Aβ42, and the ratio of Aβ40/Aβ42 among the AD, CAA, AD + CAA, and less Aβ groups were similar to those in the human brain extracts (Fig. 3i–l).

### A11-positive oligomers and HMW Aβ oligomers in the human brain extracts

**Fig. 4:**
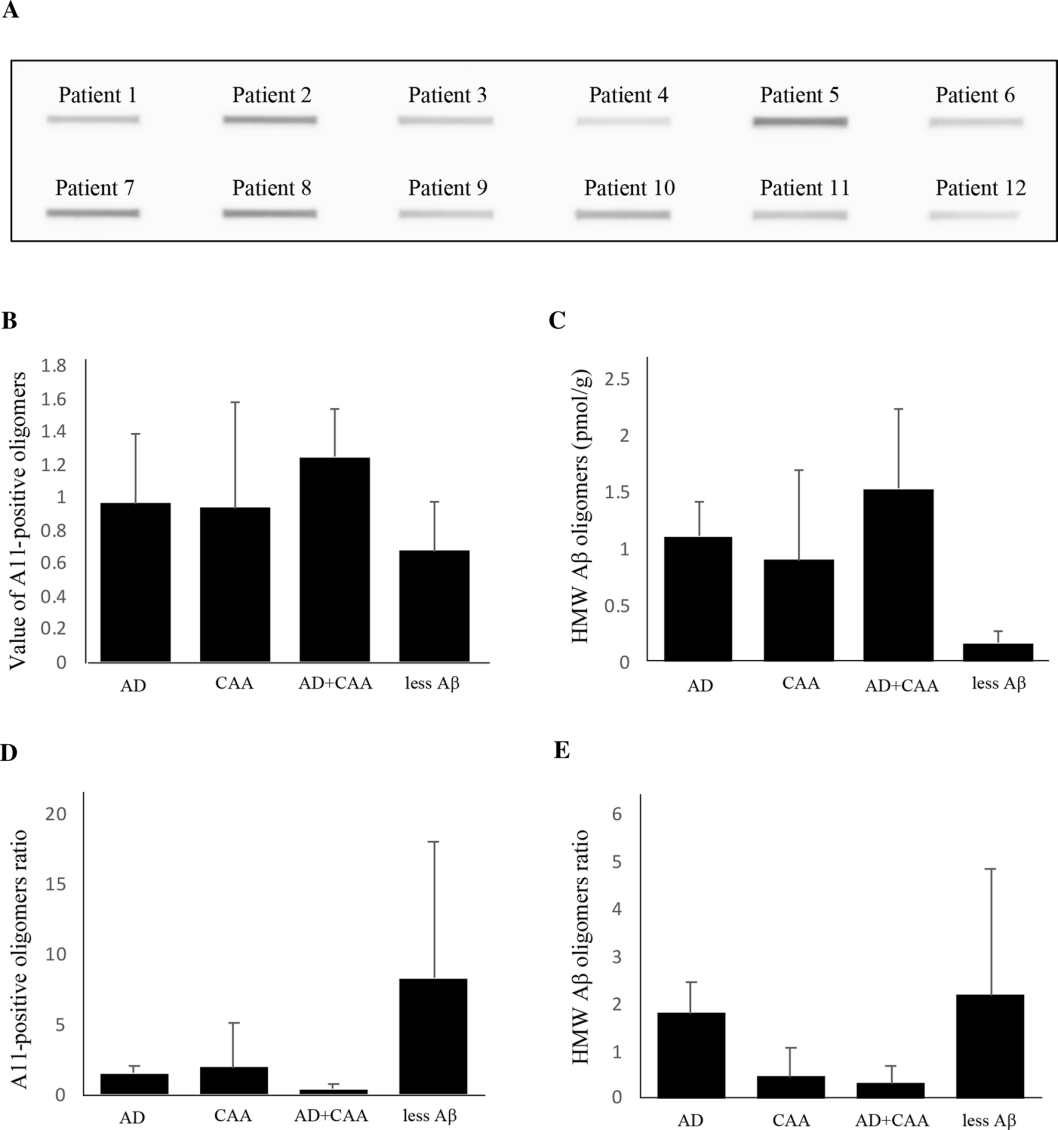
A11-positive oligomers and high molecular weight (HMW) Aβ oligomers in the human brain extracts. A11-positive oligomers for the Patient 1–12 in the Table 1 were anlayzed by dot blot analyses (**A**). Quantification of densitometry showed that the value of A11-positive oligomers in Alzheimer’s disease (AD), cerebral amyloid angiopathy (CAA), and less Aβ groups were slightly lower than those of the AD + CAA group (**B**). Similarly, the concentrations of HMW Aβ oligomers in the AD + CAA group were slightly higher than those in the other three groups (**C**). The A11-positive oligomer ratio and HMW Aβ oligomer ratio in the less Aβ group were higher than those in the other three groups (**D** and **E**)

In the analysis of A11-positive oligomers in the human brain extracts, the amount of A11-positive oligomers and the concentration of HMW Aβ oligomers in the AD + CAA group were slightly higher than in the other three groups (Fig. 4b and c). The A11-positive oligomer ratio and HMW Aβ oligomer ratio in the less Aβ group were higher than in the other three groups (Fig. 4d and e). However, statistical analyses were not performed due to the small number of the patients.

### Aβ pathology in the R1.40 APP-transgenic mice 1 year after the injection of human brain homogenates

**Fig. 5:**
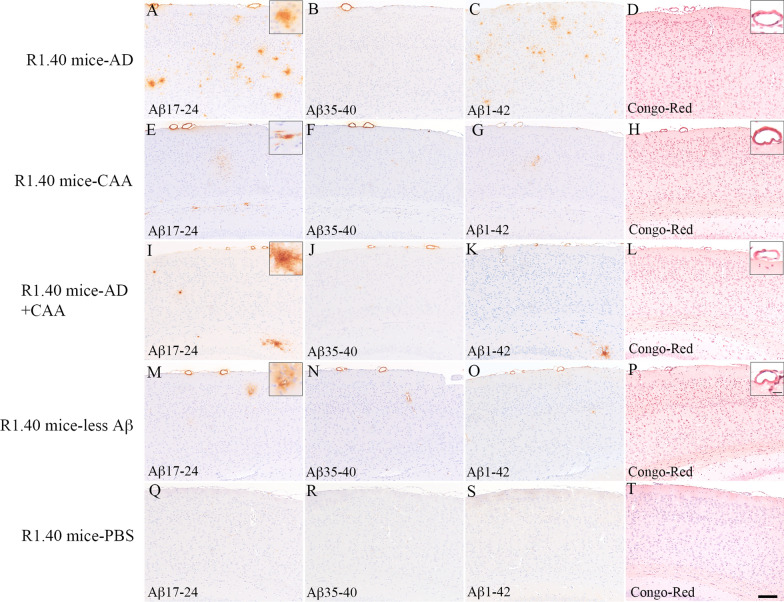
Representative immunohistochemical images of R1.40 mice injected with autopsied human brain extracts. Representative images of the R1.40 mice injected with human brain extracts from the patients with Alzheimer’s disease (AD) (**A**–**D**), cerebral amyloid angiopathy (CAA) (**E**–**H**), AD + CAA (I**–L**), and less amyloid β peptide (Aβ) pathology (**M**–**P**), and PBS (**Q**–**T**). Antibodies against Aβ_17–24_ (4G8, 1:5,000) (**A, E, I, M,**, and **Q**), Aβ_35–40_ (1A10, 1:1,000) (**B, F, J, N**, and **R**), and Aβ_1–42_ (1:100) (**C, G, K, O**, and **S**) were used as primary antibodies for immunohistochemistry, and Congo red staining was also performed (**D, H, L, P**, and **I**). Diffuse Aβ plaques, not cored plaques, and CAA were observed in all groups of mice injected with human brain extracts (**A, B, C, E, F, G, I, J, K, M, N**, and **O**). The small inserts show diffuse Aβ plaques at high magnification (**A, E, I**, and M). Vessels with CAA were stained with Congo red, but few Congo red-positive lesions were observed in the brain parenchyma (**D, H, L**, and **P**). The small inserts show Congo red-positive vessels with high magnification (**D, H, L**, and **P**). In the group of the PBS-injected mice, neither Aβ plaques nor CAA were observed (**Q**–**T**). The scale bar represents 100 µm, and 25 µm for small inserts

The Aβ pathology of the mouse brain 12 months after the injection of the human brain extracts is shown in Fig. 5. The Aβ pathology characteristics were similar in all four groups of mice injected with the human brain extracts. We found diffuse Aβ plaques, not cored plaques, and CAA both in the cortices and hippocampi of all groups of mice injected with human brain extracts (Fig. 5a–c, e–g, i–k, m–o). The vessels with CAA were stained Congo red, but few Congo red-positive lesions were observed in the brain parenchyma (Fig. 5d, h, l, and p). In PBS-injected mice, neither Aβ plaques nor CAA were observed (Fig. 5q–t), which is consistent with our previous report [19].

**Fig. 6:**
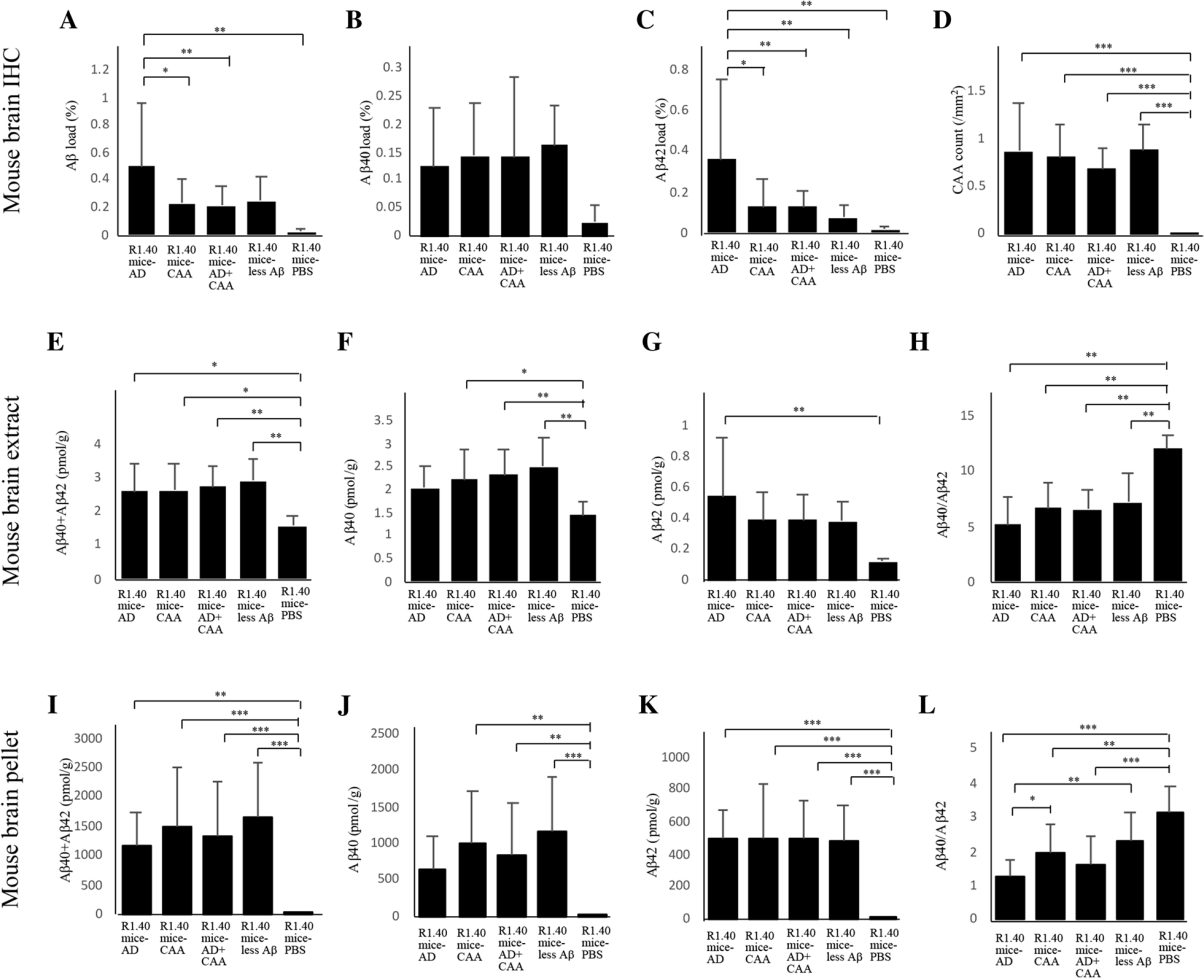
Quantitative analysis of amyloid β protein (Aβ) pathology and biochemical studies in R1.40 mice. Quantitative analysis of the Aβ pathology of the R1.40 mice injected with human brain extracts from the Alzheimer’s disease (AD) (R1.40 mice-AD), cerebral amyloid angiopathy (CAA) (R1.40 mice-CAA), AD + CAA (R1.40 mice-AD + CAA), and less Aβ pathology groups (R1.40 mice-less Aβ), and PBS (R1.40 mice-PBS) (A–D). Concentrations of Aβ40, Aβ42, and Aβ40 + Aβ42, and ratio of Aβ40/Aβ42 in the mouse brain extracts (E–H) and in the mouse brain pellets (I–L). The Aβ load in the R1.40 mice-AD was significantly higher than that in the R1.40 mice-CAA, R1.40 mice-AD + CAA, and R1.40 mice-PBS despite no significant difference in Aβ loads between R1.40-AD and R1.40 mice-less Aβ (A). The Aβ42 load was significantly higher in the R1.40 mice-AD than in all the other groups of R1.40 mice (C), while the Aββ40 load was not significantly different among them (B). CAA counts were not significantly different among the R1.40 mice-AD, R1.40 mice-CAA, R1.40 mice-AD + CAA, and R1.40 mice-less Aβ, although the CAA count in R1.40 mice-PBS was significantly lower than in all other mice groups (D). In the mouse brain extract, no significant difference in the concentrations of Aβ0, Aβ42, and Aββ40 + Aβ42 in the mouse brain extracts was observed among R1.40 mice-AD, R1.40 mice-CAA, R1.40 mice-AD + CAA, and R1.40 mice-less Aβ (E–G). Compared to R1.40 mice-PBS, concentrations of Aβ40 in R1.40 mice-CAA, R1.40 mice-AD + CAA, and R1.40 mice-less Aβ; that of Aβ42 in R1.40 mice-AD; and that of Aβ40 + Aβ42 in R1.40 mice-AD, R1.40 mice-CAA, R1.40 mice-AD + CAA, and R1.40-less Aβ were significantly higher (E–G). The Aβ40/Aβ42 ratio of R1.40 mice-PBS was significantly higher than that of R1.40 mice-AD, R1.40 mice-CAA, R1.40 mice-AD + CAA and R1.40 mice-less Aβ, although the Aβ40/Aβ42 ratio was not significantly different among the 4 groups of R1.40 mice injected with human brain extracts (H). The Aβ40/Aβ42 ratios of all groups of R1.40 mice were Aβ40 dominant (H). In the mouse brain pellets, the concentrations of Aβ40, Aβ42, or Aβ40 + Aβ42 were not significantly different among R1.40 mice-AD, R1.40 mice-CAA, R1.40 mice-AD + CAA, and R1.40 mice-less Aβ (I–K), although the concentrations of Aβ40 + Aβ42 and Aβ42 in R1.40 mice-PBS were significantly lower than those in all other groups (I and K). The concentration of Aβ40 in R1.40 mice-PBS was significantly lower than that in R1.40 mice-CAA, R1.40 mice-AD + CAA, and R1.40 mice-less Aβ (J). The Aβ40/Aβ42 ratio of R1.40 mice-PBS was significantly higher than that of R1.40 mice-AD, R1.40 mice-CAA and R1.40 mice-AD + CAA, and the Aβ40/Aβ 42 ratio of R1.40 mice-AD was significantly lower than that of R1.40 mice-CAA and R1.40 mice-less Aβ (L). The Aβ40/Aβ42 ratio of all groups of R1.40 mice indicated Aβ40 dominance (L). *p < 0.01, **p < 0.05, ***p < 0.001

The quantitative analysis of Aβ pathology in the human brain extract or PBS injected-R1.40 APP-transgenic mice is shown in Fig. 6. The Aβ load in R1.40 mice-AD (n = 22) was significantly higher than that in the R1.40 mice-CAA (n = 23), R1.40 mice-AD + CAA (n = 31), and R1.40 mice-PBS (n = 9), although there was no significant difference between R1.40 mice-AD and R1.40 mice-less Aβ (n = 14), and among R1.40 mice-CAA, R1.40 mice-AD + CAA and R1.40 mice-less Aβ (Fig. 6a). The Aβ42 load in the R1.40 mice-AD was significantly higher than that in all other groups of R1.40 mice (Fig. 6c), while the Aβ40 load was not significantly different among them (Fig. 6b). The CAA count was not significantly different among the R1.40 mice-AD, R1.40 mice-CAA, R1.40 mice-AD + CAA, and R1.40 mice-less Aβ, although the CAA count in R1.40 mice-PBS was significantly lower than that in all other mice groups (Fig. 6d).

### Concentration of Aβ in the mouse brain extracts and mouse brain pellets

There was no significant difference in the concentration of Aβ40, Aβ42, or Aβ40 + Aβ42 in the mouse brain extracts among R1.40 mice-AD, R1.40 mice-CAA, R1.40 mice-AD + CAA, and R1.40 mice-less Aβ (Fig. 6e–g). The concentration of Aβ40 + Aβ42 in R1.40 mice-PBS was significantly lower than in all other groups of R1.40 mice (Fig. 6e). The concentration of Aβ40 in R1.40 mice-PBS was significantly lower than that in R1.40 mice-CAA, R1.40 mice-AD + CAA, and R1.40 mice-less Aβ (Fig. 6f). The concentration of Aβ42 in R1.40 mice-PBS was significantly lower than that in R1.40 mice-AD (Fig. 6g). The Aβ40/Aβ42 ratio of R1.40 mice-PBS was significantly higher than that of R1.40 mice-AD, R1.40 mice-CAA, R1.40 mice-AD + CAA, and R1.40 mice-less Aβ, although the Aβ40/Aβ42 ratio was not significantly different among all four groups of human brain extract-injected R1.40 mice (Fig. 6h). The Aβ40/Aβ42 ratio of all groups of R1.40 mice indicated Aβ40 dominance (Fig. 6h).

In the mouse brain pellets, the concentrations of Aβ40, Aβ42, or Aβ40 + Aβ42 were not significantly different among R1.40 mice-AD, R1.40 mice-CAA, R1.40 mice-AD + CAA, and R1.40 mice-less Aβ (Fig. 6i–k). The concentrations of Aβ40 + Aβ42 and Aβ42 in R1.40 mice-PBS were significantly lower than those in all other groups of R1.40 mice (Fig. 6i and k). The concentration of Aβ40 in R1.40 mice-PBS was significantly lower than that in R1.40 mice-CAA, R1.40 mice-AD + CAA, and R1.40 mice-less Aβ (Fig. 6j). The Aβ40/Aββ42 ratio of R1.40 mice-PBS was significantly higher than that of R1.40 mice-AD, R1.40 mice-CAA and R1.40 mice-AD + CAA, and the Aβ40/Aβ 42 ratio of R1.40 mice-AD was significantly lower than that of R1.40 mice-CAA and R1.40 mice-less Aβ (Fig. 6l). The Aβ40/Aβ42 ratio of all groups of R1.40 mice indicated Aβ40 dominance (Fig. 6l).

### Aβ and CAA seeding activity

**Fig. 7:**
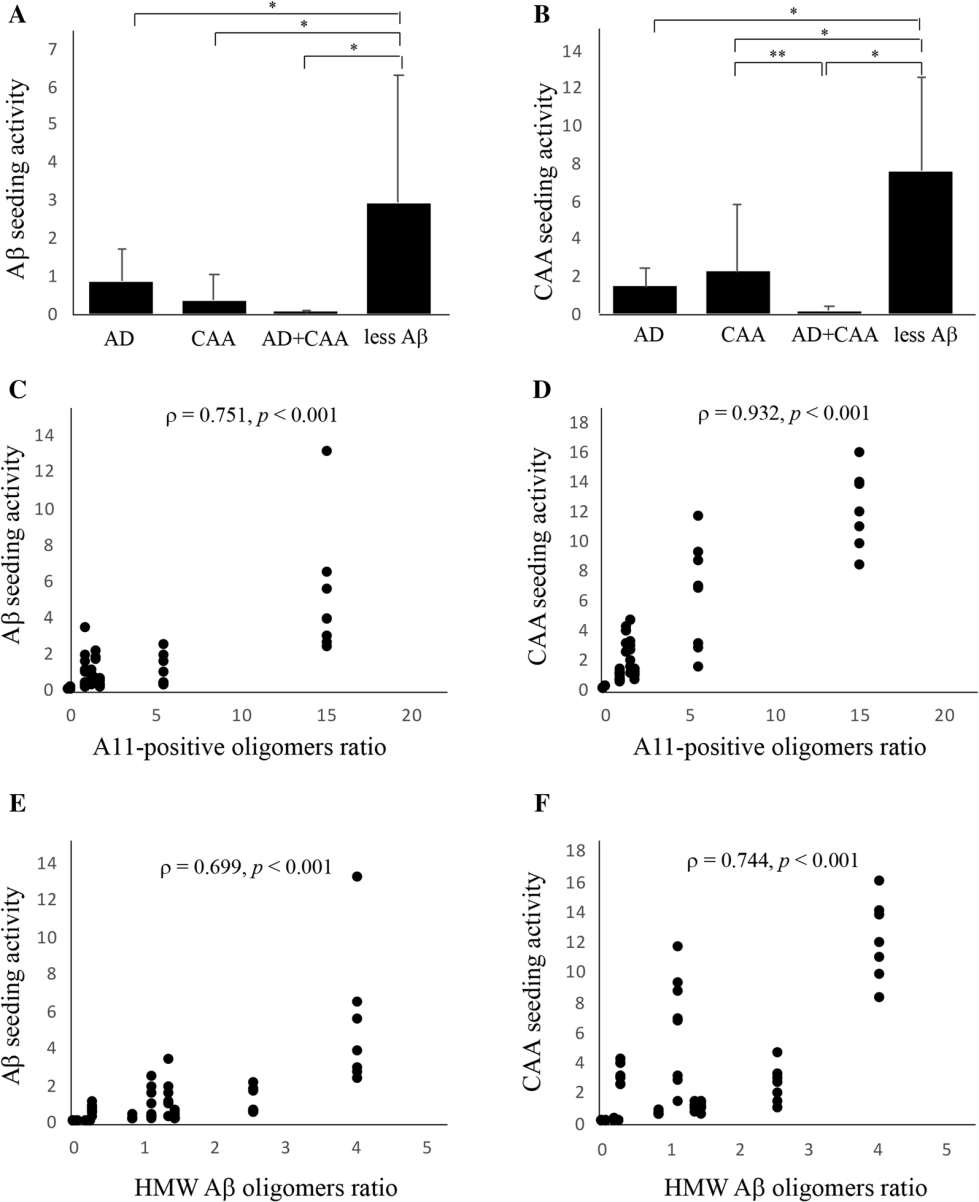
Amyloid β peptide (Aβ and cerebral amyloid angiopathy (CAA) seeding activities of the autopsied patients. Aβ and CAA seeding activities among the Alzheimer’s disease (AD), CAA, AD + CAA, and less Aβ groups (**A** and **B**), and correlations between Aβ or CAA seeding activities and A11-positive oligomer ratios or high molecular weight Aβ oligomers ratios (**C**–**F**). Aβ and CAA seeding activities in the less Aβ group were significantly higher than those in the other 3 human autopsied patient groups (**A** and **B**). In terms of CAA seeding activity, the value in the AD + CAA group was significantly lower than that in the CAA group (B). Aβ and CAA seeding activity was significantly correlated with the A11-positive oligomer ratio (**C** and** D**) and the HMW Aβ oligomer ratio (**E** and** F**)

Aβ and CAA seeding activities in the less Aβ group were significantly higher than those in the other three autopsied human patient groups (Fig. 7a and b). CAA seeding activity was significantly lower in the AD + CAA group than that in the CAA group (Fig. 7b). The A11-positive oligomer ratio and HMW Aβ oligomer ratio, which were much higher in the less Aβ group than in the other three groups (Fig. 4c and d), similar to the Aβ and CAA seeding activities, had a significant positive correlation with the A11-positive oligomer ratio and HMW Aβ oligomer ratio (Fig. 7c–f).

### PK resistance of Aβ in the human and mouse brain extracts

**Fig. 8:**
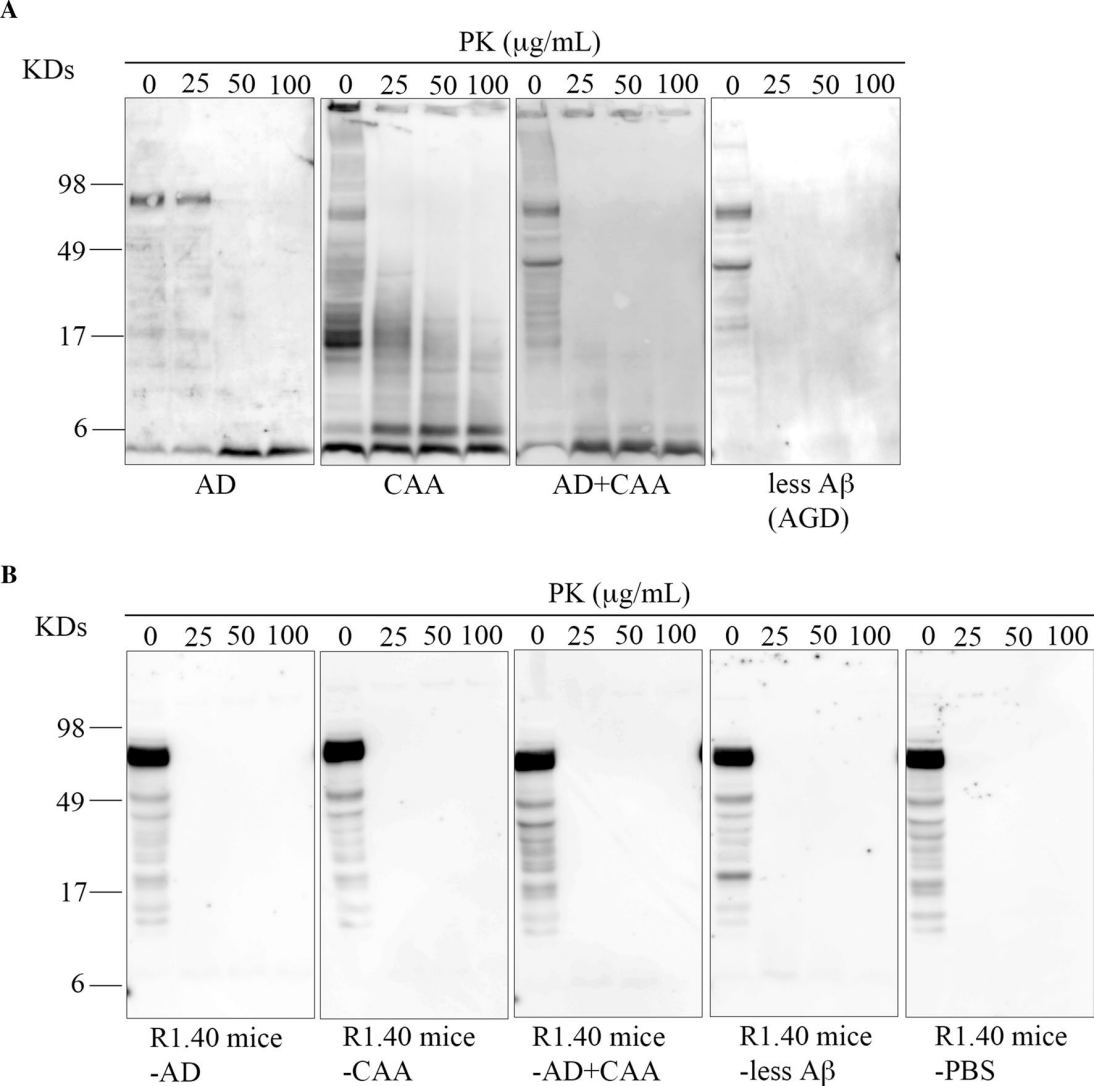
Proteinase K resistance of amyloid β peptide (Aβ) in human and mouse brain extracts. Human brain extracts of Alzheimer’s disease (AD), cerebral amyloid angiopathy (CAA), AD + CAA and less Aβ groups (**A**) as well as mouse brain extracts of R1.40 mice-AD, R1.40 mice-CAA, R1.40 mice-AD + CAA, R1.40 mice-less Aβ and R1.40 mice-PBS (**B**) were digested with 0, 25, 50, and 100 µg/mL of PK, and analyzed by Western blotting using antibodies against Aβ_1–16_ (6E10, 1:5,000) as the primary antibodies. In the human brain extracts, Aβ oligomers were digested by PK in all four groups (**A**). However, the signals of the Aβ monomers and dimers were different among the 4 groups; the signals of the Aβ monomers increased in the AD and AD + CAA groups, those of the Aβ dimers increased in the CAA group, and almost no signal of Aβ monomer and dimer signals were detected in the less Aβ group (**A**). On the other hand, in the mouse brain extracts, Aβ oligomers were digested and the signals of the Aβ dimers were lightly present (**B**)

In the human brain extracts, Aβ oligomers were digested by PK in the all four groups (Fig. 8a). However, the signals of the Aβ monomers and dimers were different among the four groups; the signals of the Aβ monomers increased in the AD and AD + CAA patient groups, those of the Aβ dimers increased in the CAA group, and almost no Aβ monomer and dimer signals were detected in the less Aβ group (Fig. 8a). On the other hand, in the mouse brain extracts, Aβ oligomers were digested and the signals of the Aβ dimer were lightly present (Fig. 8b).

## Discussion

In the present study, although the morphological features of Aβ pathology, such as cored plaques, diffuse plaques, and CAA, were clearly different among the AD, CAA, AD + CAA and less Aβ groups in the autopsied patients, those in the R1.40 mice injected with the human brain extracts were very similar among the four groups. In particular, the CAA scores of the AD and less Aβ groups were much lower than those of the CAA and AD + CAA groups, while the CAA counts of the mice injected with human brain extracts were not significantly different among the four groups. Concerning the biochemical features of Aβ, the concentrations of Aβ42 in the human brain extracts and pellets of the AD group were greater than the concentrations of Aβ40, while those in the mouse brain extracts of the R1.40 mice-AD were the opposite. Furthermore, the Aβ PK resistance of the human brain extracts was different among the different patient groups, which might be due to differences in the Aβ strain, while the PK resistance in the mouse brain extracts was not different among the groups. These results indicated that the pathological and biochemical features of Aβ in the autopsied human brains were not preserved in the R1.40 mice injected with human brain extracsts, which was contrary to the Aβ strain hypothesis. Recently, several studies have shown that Aβ fibrils from patients with AD have several structural variations [38, 50], and that the diversity of Aβ pathology in mouse models and human brains might be dependent on different molecular architectures of Aβ aggregates, which are the so-called Aβ strains that are similar to prion strains [24, 45, 51, 56, 60]. In prion diseases, several different types of PrP^Sc^ with different neuropathological and biochemical profiles despite identical PrP amino acid sequences have been reported, and the diversity of PrP^Sc^ reflects differences in PrP^Sc^ conformations, which are called prion strains [23, 48]. Furthermore, there have been several types of prion diseases with different characteristics of clinical and neuropathological features, and these differences are influenced by prion strains and genetic variations of the PrP gene [23, 48]. Previous studies using animal models or patients with acquired prion diseases have shown that neuropathological and biochemical features of PrP^Sc^ in donor subjects are preserved in host subjects, or novel prion strains with characteristics of both the donors and hosts have emerged [23, 48]. However, the results of the present study showed that Aβ deposition in brain parenchyma or blood vessels was not defined by Aβ strain-specific information, suggesting the importance of Aβ strains in the host or in Aβ production and clearance.

In the present study, the CAA counts in the R1.40 mice injected with the human brain extracts from all four groups, including R1.40 mice-AD and -less Aββ, were significantly higher than those in the R1.40 mice-PBS, although CAA was hardly detected in the patients with AD and less Aβ groups. On the other hand, regarding parenchymal Aβ deposition in the R1.40 mice, Aβ load was not different among the R1.40 mice injected with the human brain extract and PBS, except for significantly higher Aβ and Aβ42 loads in R1.40 mice-AD. Previous studies have shown that both parenchymal and vascular Aβ depositions were observed in aged R1.40 mice, without obvious differences in the severity of Aβ plaque accumulation and CAA [39]. Several pathological studies using autopsied patients with iatrogenic Creutzfeldt–Jakob disease (CJD) showed that Aβ pathology might be transmitted from human to human through medical procedures, including intramuscular injection of growth hormones derived from cadaveric human pituitary glands and cadaveric dura mater grafting [8, 13, 16, 22, 30, 53]. In cases of transmission of Aβ pathology in humans, incidental Aβ pathology recognized in patients with iatrogenic CJD was predominantly observed in blood vessels [8, 64]. Furthermore, most patients who developed CAA-related haemorrhage at a younger age had histories of neurosurgery with or without evidence of cadaveric dura mater grafting [4, 9, 14, 20, 25, 31, 47, 49, 62, 64]. The results of the present study and the previously reported data on iatrogenic human-to-human transmission of Aβ pathology show that Aβ fibrils aggregated by exogenous Aβ seeds might deposit on vessels rather than the brain parenchyma, regardless of the Aβ strain.

Interestingly, in the present study, parenchymal and vascular Aβ depositions were observed in the R1.40 mice-less Aβ group, although there were only a few diffuse plaques and CAA in the patients in the less Aβ group. In particular, Aβ and CAA seeding activities in the less Aβ group were significantly higher than they were in the other three groups. A previous study estimated the time course of Aβ seeding activity in an AD mouse model and showed that Aβ seeding activity peaked sharply during the initial phase of Aβ deposition [67], which was consistent with the results of the patients in the less Aβ group in the present study, which should be in the initial phase of Aβ deposition in the brain. Furthermore, another previous study showed that brain homogenates of pre-symptomatic or asymptomatic stages of AD accelerated Aβ deposition in vivo [12]. In brain homogenates, soluble forms of Aβ, such as monomers and oligomers, as well as Aβ fibrils, play important roles in Aβ seeding [15, 35, 41]. A recent study reported the importance of Aβ oligomers in the early and rapid seed-induced aggregation process as the first nucleation step in the absence of Aβ fibrils in the brains of AD mouse models [35]. In the present study, the A11-positive oligomer and HMW Aβ oligomer ratios in the less Aβ group were higher than they were in the other groups, and they had a significant positive correlation with Aβ and CAA seeding activities. These results suggest that Aβ oligomers play important roles in the initial phase of Aβ deposition, and that the ratio of Aβ oligomers increases in the brain during the early phase of Aβ deposition in humans. Furthermore, our results indicate that Aβ pathology may be transmitted from patients with little Aβ pathology via neurosurgical procedures. Thus, the development of inactivation methods for Aβ seeding activity to prevent iatrogenic transmission of Aβ pathology is urgently required.

In the present study, the *Apo E* genotype of autopsied patients was ε3/3 in seven patients (one in the CAA group, four in the AD + CAA group, and two in the less Aβ group), ε3/4 was in two patients (one in the AD group and another in the CAA group), and ε2/3 in a patient in the CAA group. We found no influence of the *Apo E* genotype on the pathological and biochemical features of Aβ in the autopsied brain-injected R1.40 mice. We did not examine APP, preseniline (PS) 1 and PS 2 genes in all of the autopsied patients; however we believe that all patients did not have mutations in these genes because they were older and had no family history of AD.

In conclusion, exogenous Aβ seeds from different Aβ pathologies induced Aβ deposition in the blood vessels rather than the brain parenchyma without being influenced by Aβ strain-specific information, which might be a reason why CAA is a predominant feature of Aβ pathology in iatrogenic transmission cases. Soluble Aβ oligomers in the human brain play important roles in Aβ transmission, and iatrogenic transmission of Aβ pathology might occur in patients with little Aβ pathology in the brain.

## Data Availability

The data that support the findings of this study are available from the corresponding author upon reasonable request.

## Abbreviations

AD: Alzheimer’s disease
Aβ: Amyloid β peptide
NFTs: Neurofibrillary tangles
CAA: Cerebral amyloid angiopathy
Aβ42: Aβ with 42 amino acid peptides
Aβ40: Aβ with 40 amino acid peptides
IPAD: Intramural peri-arterial drainage
PrP: Prion protein
Abnormal PrP: PrP^Sc^
APP: Amyloid precursor protein
HCHWA: Hereditary cerebral haemorrhage with amyloidosis
*APOE*: Apolipoprotein E gene
CERAD: Consortium to Establish a Registry for AD
R1.40 mice: Homozygous R1.40 APP-transgenic mice
ELISA: Enzyme-linked immunosorbent assays
A11-positive oligomer ratio: The value of A11-positive oligomers was divided by the concentration of Aβ40 + Aβ42
HMW: High molecular weight
HMW Aβ oligomers ratio: The concentration of HMW-positive oligomers was divided by the concentration of Aβ40 + Aβ42
PK: Proteinase K
R1.40 mice-AD: R1.40 mice injected with human brain extract from the patients in the AD group
R1.40 mice-CAA: R1.40 mice injected with human brain extract from the patients in the CAA group
R1.40 mice-AD + CAA: R1.40 mice injected with human brain extract from the patients in the AD + CAA group
R1.40 mice-less Aβ: R1.40 mice injected with human brain extract from the patients in the less Aβ group
R1.40 mice-PBS: R1.40 mice injected with PBS
AGD: Argyrophilic grain disease
PS: Preseniline
